# Dysfunctional attributions of success as a distinct feature of amotivation

**DOI:** 10.1038/s41537-024-00441-9

**Published:** 2024-02-12

**Authors:** Alisa L. A. Schormann, Katja Butschbach, Tania M. Lincoln, Marcel Riehle

**Affiliations:** Clinical Psychology and Psychotherapy, Institute of Psychology, Faculty of Psychology and Human Movement, Universität Hamburg, Hamburg, Germany

**Keywords:** Schizophrenia, Psychosis

## Abstract

We examined the association between causal attributions and self-reported motivational negative symptoms (amotivation) in a German online community sample (*n* = 251). Bivariate correlations revealed significant associations between amotivation and attribution of success to external, variable, and specific causes. No associations between amotivation and failure attributions were found. Our data suggest that demotivational causal attributions of success could be a feature of amotivation and a promising target for research and intervention.

Demotivating beliefs play a crucial role in amotivation, a core aspect of many forms of psychopathology, most notably as a negative symptom of schizophrenia^1,2^. As a negative symptom in schizophrenia it represents the extreme end of a continuum that can be observed in the general population^3–5^ and that has a dimensional trait-like component (i.e., as part of negative schizotypy). Cognitive models assume that demotivating beliefs form in response to repeated experiences of failure^1^. However, little is known about *how* this process unfolds. We propose that causal attributions, defined as the explanations one has regarding the cause of past events^6^, are an important explanatory factor for *how* experiences transform into demotivating beliefs. More specifically, when a person tends to attribute failure to internal, stable, or global causes and success to external, variable, or specific causes, demotivating beliefs (e.g., “If I fail at my work, then I am a failure as a person.”) are a likely consequence. A tendency to internalize failure and to externalize success was found in association with elevated negative symptoms in schizophrenia^7^. However, no study so far has investigated the putative relationship of amotivation and the attributional dimensions stability and globality.

We hypothesized that (a) attributing failure to internal, stable, and global causes and (b) attributing success to external, variable, and specific causes is associated with higher levels of amotivation (i.e., low experienced and anticipated pleasure and low motivation for social, recreational, and vocational activities).

We analyzed the associations between different attributional styles (Attributional Style Questionnaire (ASQ^8^), self-reported positive (Community Assessment of Psychic Experiences, positive symptoms, CAPE-POS^4^) and depressive symptoms (Center for Epidemiological Studies—Depression Scale, CES-D^9^) and amotivation (Motivation and Anticipation of Pleasure—Self Report, MAP-SR^10^) in correlation analyses. The data came from a convenience online community sample (*n* = 251; see Table 1 for sample characteristics, Table 2 for correlation analyses and supplemental material for trial flow chart [Supplemental Fig. S1], data cleansing procedures, and additional sample characteristics [Supplemental Table S1], as well as for results and a supplementary discussion of multiple regression analyses for each attributional variable included as predictor alongside depression and positive symptoms as additional covariates and with amotivation as dependent variable [Supplemental Table S2]). For all analyses the Bonferroni corrected *p*-value of 0.00064 has been used to account for family-wise error accumulation.

**Table 1:** Sample characteristics.

Variable		*M* or %	*SD*	Range
Gender				
Female		70.1%	—	—
Male		29.5%	—	—
Diverse		0.4%	—	—
Age (in years)		31.87	12.05	19–72
Lifetime diagnosis of mental illness				
No diagnosis		68.9%	—	—
Yes		20.3%	—	—
Yes, but do not know diagnosis		4.0%	—	—
No answer		6.8%	—	—
Highest degree of education				
University entrance diploma		27.1%	—	—
Academic degree (bachelor, master, PhD)		57.4%	—	—
Other		15.5%	—	—
Recruitment way				
Students of Universität Hamburg		2.0%	—	—
External students via poll-pool.com, thesius.de, surveycircle.com		53.8%	—	—
Microworker via figure-eight		14.7%	—	—
Other		29.5%	—	—
MAP-SR		19.90	7.81	0–54
CES-D		12.28	8.61	0–42
CAPE-POS		28.12	5.72	20–52
ASQ-success	Internality	76.06	12.32	34–106
	Stability	79.24	10.44	53–106
	Globality	79.21	14.68	30–112
ASQ-failure	Internality	67.35	12.74	29–111
	Stability	66.16	14.22	21–108
	Globality	60.61	16.07	17–111

*Note. n* = 251.

*CES-D* Center for Epidemiologic Studies-Depression Scale, *CAPE-POS* Community Assessment of Psychic Experiences, positive symptoms subscale, *MAP-SR* motivation and pleasure scale-self report, *ASQ* Attributional Style Questionnaire. Percentage data does not add up to 100% due to rounding.

**Table 2:** Bivariate correlations between all variables.

		Gender^a^	Education^b^	Mental disorder diagnosis^c^	Age	MAP-SR	CES-D	CAPE-POS	ASQ-success			ASQ-failure		
									Internality	Stability	Globality	Internality	Stability	Globality
Education^b^		0.046^d^	—											
Mental disorder diagnosis^c^		0.057^d^	−0.153^d^	—										
Age		−0.070^e^	−0.339*^e^	−0.027^e^	—									
MAP-SR		0.030^e^	−0.096^e^	0.191^e^	−0.010 ^f^	—								
CES-D		0.139^e^	0.033^e^	0.283*^e^	−0.167 ^f^	0.527*^f^	—							
CAPE-POS		0.029^e^	−0.001^e^	0.073^e^	−0.097 ^f^	0.146 ^f^	0.346*^f^	—						
ASQ-success	Internality	−0.086^e^	0.036^e^	−0.231*^e^	−0.045 ^f^	−0.370*^f^	−0.219*^f^	−0.081 ^f^	—					
	Stability	−0.165^e^	0.035^e^	−0.048^e^	0.055 ^f^	−0.270*^f^	−0.205 ^f^	−0.124 ^f^	0.464*^f^	—				
	Globality	−0.133^e^	−0.005^e^	−0.127^e^	0.051 ^f^	−0.278*^f^	−0.131 ^f^	0.043 ^f^	0.542*^f^	0.336*^f^	—			
ASQ-failure	Internality	0.000^e^	−0.088^e^	0.132^e^	−0.093 ^**f**^	−0.003 ^f^	0.182 ^f^	0.070 ^f^	0.073 ^f^	−0.098 ^f^	−0.038 ^f^	—		
	Stability	−0.138^e^	−0.064^e^	0.237*^e^	−0.004 ^f^	0.146 ^f^	0.177 ^f^	0.077 ^f^	−0.194 ^f^	0.180 ^f^	−0.203 ^f^	0.385*^f^	—	
	Globality	−0.131^e^	−0.014^e^	0.150^e^	−0.095 ^f^	0.101 ^f^	0.235*^f^	0.227*^f^	−0.092 ^f^	−0.105 ^f^	0.204 ^f^	0.493*^f^	0.516*^f^	—

*Note. n* = 251. Bivariate correlation coefficients and their significance with regard to variables’ individual level of measurement.

*MAP-SR* Motivation and Pleasure Scale-Self Report. *CES-D* Center for Epidemiologic Studies-Depression Scale, *CAPE-POS* Community Assessment of Psychic Experiences, positive symptoms subscale. *ASQ* Attributional Style Questionnaire.

^a^gender (male/female), diverse person excluded (*n* = 250). ^b^education (low/high). ^c^mental disorder diagnosis (no/yes). ^d^φ-coefficient for two dichotomous categorial variables. ^e^point biserial correlation coefficient for a dichotomous categorial variable and a continuous variable. ^f^Pearson correlation coefficient for two continuous variables. For the interpretation of the correlations, the Bonferroni corrected level of significance 0.05/78 = 0.00064 has been implemented.

* p < 0.00064.

There was no significant association between higher levels of amotivation and the attribution of failure to internal (*r* = -0.003, *p* = 0.9670), stable (*r* = 0.146, *p* = 0.0205), or global causes (*r* = 0.101, *p* = 0.1099). Higher levels of amotivation were significantly associated with the attribution of success to less internal (*r* = -0.370, *p* < 0.0001), less stable (*r* = -0.270, *p* < 0.0001), and less global causes (*r* = -0.278, *p* < 0.0001). These results were robust against additionally controlling for depression and positive symptoms in regression models (see Table S2 in the supplemental material). All effect sizes are classified as small to moderate^11^.

There was no significant association between amotivation and positive symptoms (*r* = 0.146, *p* = 0.0206). Higher levels of positive symptoms were significantly correlated with attributing failure to more global causes (*r* = 0.227, *p* = 0.0003) with a small to moderate effect size^11^, but not with any other attributional style. Higher levels of depressive symptoms were significantly associated with the attribution of success to less internal (*r* = -0.219, *p* = 0.0005) causes and with the attribution of failure to more global causes (*r* = 0.235, *p* = 0.0002), each with a small to moderate effect sizes^11^. There were no significant associations between symptoms of depression and any other attributional style. It needs noting that higher levels of amotivation were significantly correlated with higher levels of depressive symptoms (*r* = 0.527, *p* < 0.0001) with a large effect size^11^.

Our data thus do not fully confirm our hypotheses derived from cognitive models of negative symptoms^1,2^ and earlier findings^7^ which had suggested that a particular attributional style for failure was related to amotivation. Instead, our data suggest that global attributions of failure are more specific to depressive symptoms, matching long-known attributional tendencies in depression^12^.

Higher levels of amotivation were, however, associated with attributing success to external, stable, and global causes. Accordingly, people who view achieving positive outcomes as unattached to their own actions, or as an outlier that is not predictive of future events or related to outcomes of similar events, may be more likely to develop motivational problems. This type of attribution prevents successes from challenging pre-existing demotivating beliefs, such as low expectations for success^1,2^, and can explain why people with motivational negative symptoms show inhibited learning from positive outcomes^13^. Against our theoretical assumptions, amotivation was not associated with any attributional styles of failure.

This is the first study to specifically target associations between the attributional dimensions of internality, stability, and globality and amotivation. To corroborate our findings of dysfunctional external, variable, and specific success attributions in amotivation, future research needs to address the limitations of our study (i.e., sample demographics are not representative of schizophrenia samples; assessing amotivation via self-report and not via expert rating, with the MAP-SR having disputed construct validity regarding amotivation; cross-sectional design). Moreover, particularly in community samples, other aspects of psychopathology are known to overlap with self-reported negative symptoms (e.g., depression^14^). Given that, in our data depression and amotivation were highly correlated and can thus not be distinguished sufficiently, a replication in clinical samples may help disentangle the differential contributions of these two aspects of psychopathology. Further research is also required to investigate whether dysfunctional success attributions play a causal role in the formation and maintenance of clinical motivational negative symptoms. If this were the case, reattribution training specifically for strengthening internal, stable, and global attributions of success could become a promising addition to cognitive interventions for motivational negative symptoms to reinforce people’s sense of agency.

## Methods

This online study was implemented from 06/2019 to 07/2019 in EFS Survey (Questback GmbH, 2017) and approved by the local ethics committee of Universität Hamburg (AZ: 2019_232_Butschbach). Participants were recruited from the community via leaflets and a variety of internet platforms (see Table 1 and supplemental material for detailed information on recruitment). The only inclusion criterion was an age of at least 18 years. The online survey took about 30 min and included written informed consent, assessments of sociodemographic variables, symptom questionnaires (MAP-SR, CAPE-POS, CES-D), and the ASQ. The ASQ requires participants to formulate a causal explanation for hypothetical positive and negative situations in a free-text field and rate it on the dimensions internal vs. external, stable vs. variable, and global vs. specific with two items each. The data were analyzed with a series of Bonferroni-corrected correlation analyses. See the supplement for further information on the instruments used.

### Supplementary information

Supplemental Material

## Data Availability

All research data and the codebook needed to reproduce the analyses and findings of this study are publicly available for non-commercial scientific use at 10.23668/psycharchives.14147.
