# Supplementary material for: Dysfunctional attributions of success as a distinct feature of amotivation
Source: Schizophrenia (Heidelb). 2024 Feb 12;10(1):15. doi: 10.1038/s41537-024-00441-9 (PMC10861587; doi:10.1038/s41537-024-00441-9)
Supplement: Supplementary file 1 — Supplemental Material [file 41537_2024_441_MOESM1_ESM.docx]

**Supplemental Material for**

**“Dysfunctional attributions of success as a distinct feature of amotivation”**

Alisa L. A. Schormann, Katja Butschbach, Tania M. Lincoln, & Marcel Riehle
Clinical Psychology & Psychotherapy, Institute for Psychology, University of Hamburg,
Hamburg, Germany

Correspondence should be addressed to:

Alisa L.A. Schormann, [alisa.schormann@uni-hamburg.de](mailto:alisa.schormann@uni-hamburg.de)

**Table of Contents**

[**Figure S1** Participant flow in the study 3](#_Toc144285452)

[**Table S1** Detailed information on mental disorder diagnoses 4](#_Toc144285454)

[**Additional information on recruitment** 5](#_Toc144285456)

[**Additional information on instruments** 5-](#_Toc144285457)7

[**Detailed description of statistical analyses** 7-8](#_Toc144285460)

[**Data cleansing** 7](#_Toc144285461)

[**Bivariate correlations**](#_Toc144285462) 8

**Additional multiple linear regression analyses** [8-9](#_Toc144285460)

[**Table S2** Summary of the multiple regression analyses 10-11](#_Toc144285460)

# **Figure S1**

# Participant flow in the study


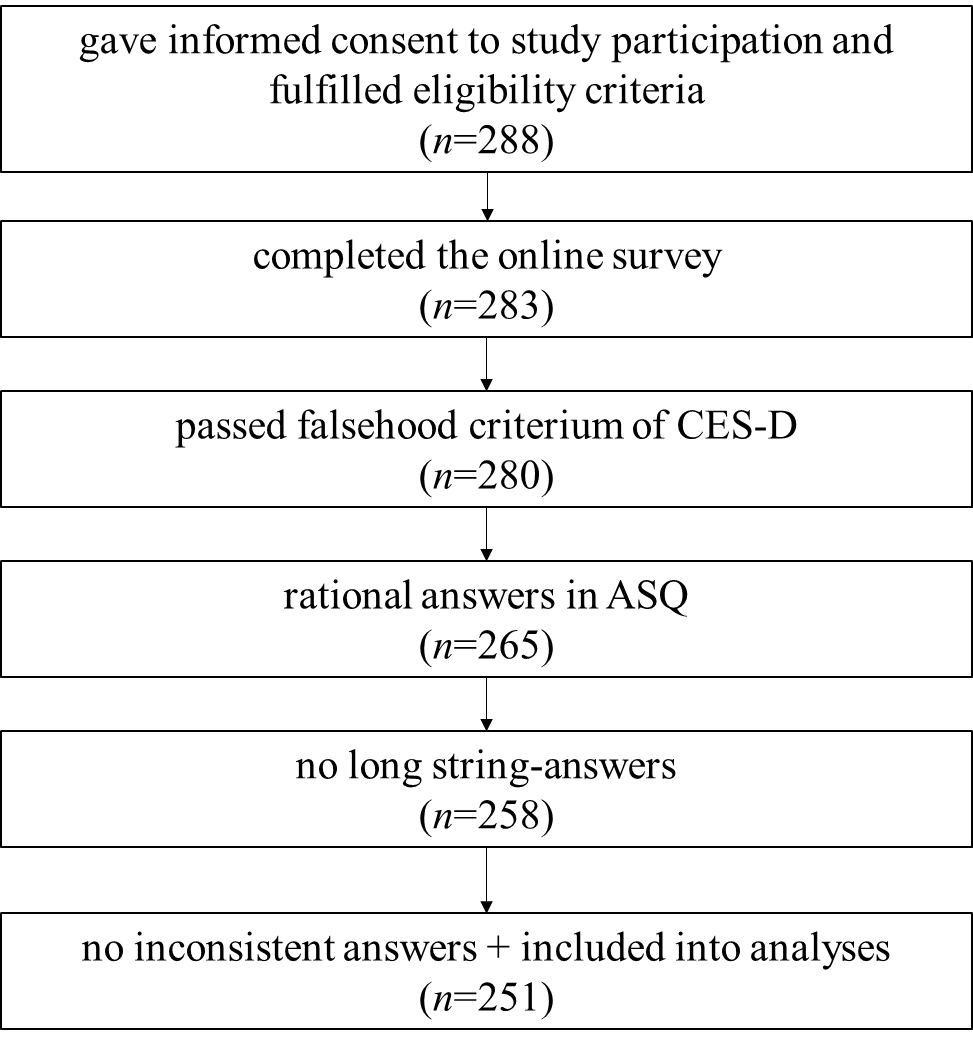


# **Table S1**

# *Depiction of open-text answers of those 20.3% (n=51) who have received a mental disorder diagnosis and gave information on the diagnosis*

|  | amount | % of participants |
| --- | --- | --- |
| depression | 26 | 50.98 |
| bipolar disorder | 2 | 3.92 |
| adjustment disorder | 7 | 13.73 |
| posttraumatic stress disorder | 8 | 15.69 |
| anxiety disorder | 9 | 17.65 |
| no further details | 4 | 7.84 |
| panic attacks | 1 | 1.96 |
| social phobias | 3 | 5.88 |
| generalized anxiety disorder | 1 | 1.96 |
| obsessive-compulsive disorder | 2 | 3.92 |
| somatoform disorder | 1 | 1.96 |
| substance use disorder | 1 | 1.96 |
| attention deficit disorder | 2 | 3.92 |
| autism spectrum disorder | 2 | 3.92 |
| eating disorder | 7 | 13.73 |
| no further details | 5 | 9.80 |
| eating disorder, not otherwise specified | 1 | 1.96 |
| bulimia nervosa | 1 | 1.96 |
| borderline personality disorder | 1 | 1.96 |
| schizophrenia | 1 | 1.96 |
| other (ambiguous wording) | 4 | 7.84 |

*Note.* *n*=51. Multiple entries per person were allowed. Total number of entries = 73. Percentage data does not add up to 100% due to rounding.

# **Additional information on recruitment**

Participants were recruited as a convenience sample using different recruitment ways to ensure for a preferably heterogenous sample. Students of Universität Hamburg were invited to participate via the university’s recruitment system SONA receiving course credit for participation. Also, external online-platforms for students were used, namely poll-pool.com, thesius.de, and surveycircle.com. On these platforms, students can promote their own online surveys by taking part in each other’s research. The microworker platform “figure-eight.com” was used additionally. Further, the online platforms facebook.com, twitter.com, ebay-kleinanzeigen.de, and psychologie-heute.de were used.

In addition to online-recruitment, leaflets were distributed in public spaces, e.g., at Universität Hamburg, supermarkets, shops, and on crossroads to invite participants. The study was advertised to participants by the following text: “What’s the matter? On the scent of the causes. Exciting online study with voucher lottery. What is it [the study] about? What causes do you attribute to the events in your life? The Clinical Psychology department at the Universität Hamburg is using an online questionnaire study to research causal attributions in connection with various psychological variables. Participation takes about 30 minutes.” (original German text: „Spannende Onlinestudie mit Gutscheinverlosung. Worum geht es? Welche Ursachen schreiben Sie den Ereignissen in Ihrem Leben zu? Die Klinische Psychologie der Universität Hamburg erforscht mit einer Onlinefragebogenstudie Ursachenzuschreibungen in Zusammenhang mit verschiedenen psychologischen Variablen. Die Durchführung nimmt ca. 30 Minuten in Anspruch.”).

Participants could choose between different ways of compensation for study participation (course credit for students of Universität Hamburg; monetary compensation of 1,80€ for microworker; code for platform-specific compensation of poll-pool.com, thesius.de, or suvreycircle.com; taking part in a lottery to win a voucher of avocadostore.de of 120€ in total: 1 voucher of 50€, two vouchers of 25€, two vouchers of 10€ for remaining participants).

# **Additional information on instruments**

The online survey included the following self-report assessments: 1) questionnaire on sociodemographic variables, 2) the Motivation and Anticipation of Pleasure – Self Report (MAP-SR; (1)), 3) the Attributional Style Questionnaire (ASQ; (2,3)), 4) the subscale for positive symptoms of the Community Assessment of Psychic Experiences, positive symptoms (CAPE-POS; (4)), and 5) the Center for Epidemiological Studies – Depression Scale (CES-D; (5)).

The questionnaire on sociodemographic variables consists of items to assess age, gender, highest educational degree, as well as prior and recent diagnosis of psychiatric disorders.

The 15 item MAP-SR assesses motivational negative symptoms. For each item, participants rate the intensity and frequency of pleasure experienced for certain situations (i.e., activities with other people, leisure activities, work activities) as well as the expected pleasure for those kinds of activities in the following weeks. Participants are asked to rate the importance of these relationships and activities as well as the level of motivation and the extent of effort to engage in these activities. Items are rated on a 5-point Likert scale from 0 (e.g., no pleasure) to 4 (e.g., very much pleasure). Sum scores of all items inverted can range between 0 and 60 points with higher scores indicating greater motivational negative symptoms.

The ASQ assesses causal attribution for eight positive and eight negative performance-related or interpersonal events, e.g., becoming wealthy or getting negative reactions for a presentation. Participants are asked to give a text explanation on their perceived major cause of the event. In a next step, they are asked to rate this cause with two items for each of the three attribution dimensions: internality (vs. externality), stability (vs. variability), and globality (vs. specificity). The dimensions are rated on a 7-point Likert scale from 1 (e.g., [the major cause of this event…] is entirely in other people or the circumstances) to 7 ([the major cause of this event…] is entirely in myself) for externality vs. internality, from 1 ([the major cause of this event…] will never again influence whether I become wealthy in the future) to 7 ([the major cause of this event…] will continue to influence whether I become wealthy in the future) for variability vs. stability, and from 1 ([the major cause of this event…] only influences whether I become wealthy) to 7 ([the major cause of this event…] also positively influences many other areas of my life) for specificity vs. globality. Higher values ​​are interpreted towards increasing internality, stability, and globality. Accordingly, lower scores point towards externality, instability, and specificity. Sum scores for internality, stability, and globality are calculated for positive and negative events each and can range between 16 and 112 points, respectively.

The 20 item CAPE positive symptoms subscale assesses psychotic experiences in the general population. For each item, participants rate the lifetime frequency with which they have experienced the described phenomenon (e.g., “Do you ever hear voices when you are alone?”) on a 4-point Likert scale from 0 (never) to 3 (almost always). Sum scores can range between 0 and 60 points with higher scores indicating greater positive symptoms.

The 15 item CES-D assesses depressive symptoms. For each item, participants rate the frequency with which they experience the described depressive symptom on a 4-point Likert scale from 0 (rarely or none of the time) to 3 (most or almost all the time). The CES-D contains two inverted items which can be used to calculate an indicator of falsehood. Sum scores can range between 0 and 45 points with higher scores indicating greater depressive symptoms.

**Detailed description of statistical analyses**

All analyses were performed using IBM SPSS Statistics (version 29.0.0).

**Data cleansing**. Data cleansing was conducted as depicted in the trial flow chart (supplementary figure S1) excluding those who 1) did not complete the survey. Further, 2) those who did not pass the falsehood criterium of the CES-D were excluded. The CES-D falsehood criterium detects stereotypical response patterns (e.g., people answering all items with similar ratings) by using the two inverted items number 9 and number 12 to test people’s response pattern, i.e., by calculating: sum of responses to not inverted items - 6,5 x sum of inverted items. The critical value for detection of stereotypical response patterns is < - 24. Next, 3) those were excluded who did not answer the ASQ free text format according to the task (e.g., typing in random letters or not giving a causal explanation but a reaction to the situation, for example “that’s nice”) more than twice (according to the ASQ manual). In a next step, 4) those were excluded who gave long string answers in the ASQ (same numbers in a row for one situational vignette). Finally, 5) those were excluded who did not answer the ASQ consistently. To identify those inconsistencies, the responses to the ASQ items for success were examined. Internality, stability, and globality are each assessed by two items per situation, so the response to item 1 was compared to the response to item 2 of the same vignette. Cases were excluded if the resulting correlation was negative.

We then conducted descriptive analyses of the sample.

## **Bivariate correlations**

For further analyses, the categorial variable *education* was reduced to two categories (“high”/ “low”) by summarizing the following answers “no degree”, “Hauptschulabschluss/ main school degree”, and “Realschulabschluss/ middle school degree” as “low education”. The answers “Fachhochschulreife/ university of applied sciences entrance qualification”, “Abitur/university entrance diploma”, “bachelor’s degree”, “master’s degree”, and “doctoral degree or higher” were summarized as “high education”. Different correlation coefficients were used according to level of measurement of the variables (see Table 2).

## **Additional multiple linear regression analyses**

**Methods.** We conducted six additional multiple linear regression analyses for each attributional style with amotivation as dependent variable, respectively. The variables relevant in terms of content or statistical correlation with amotivation, i.e., CES-D and CAPE-POS, were implemented into the multiple regression analyses as additional predictors for each analysis.

**Results.** Results of the multiple linear regression analyses are shown in supplemental Table S2. Neither attributing failure to internal (*β* = -.101, 95% *CI* [-.128, .004], *t* = -1.860, *p* = .064, *R_I_²*= .010), stable (*β* = .055, 95% *CI* [-.029, .090], *t* = 1.013, *p* = .312, *R_I_²*= .003), nor global causes (*β* = -.018, 95% *CI* [-.063, .045], *t* = -.327, *p* = .744, *R_I_²*= .000) significantly predicted amotivation.

Attributing success to external (*β* = -.268, 95% *CI* [-.236, -.104], *t* = -5.085, *p* < .001, *R_I_²*= .068), variable (*β* = -.171, 95% *CI* [-.208, -.048], *t* = -3.162, *p* = .002, *R_I_²*= .028), and specific causes (*β* = -.211, 95% *CI* [-.168, -.057], *t* = -3.976, *p* < .001, *R_I_²*= .043) significantly predicted amotivation with small to moderate effect sizes.

There was no significant association between positive symptoms and amotivation in any of the six regression analyses. Depressive symptoms, however, significantly predicted amotivation in each of the six regression analyses with *R_I_²* ranging from .197 to .268 which equals moderate to high effect sizes.

The results of these additional regression analyses match the results of our bivariate correlation analyses and add information on the influence of each attributional variable while controlling for the influences of depressive and positive symptoms.

**Table S2**

*Summary of the results of the multiple linear regression analyses for each attributional style with amotivation as dependent variable.*

|  | Dependent variable: amotivation | | | | | |
| --- | --- | --- | --- | --- | --- | --- |
|  | success attributions | | | failure attributions | | |
|  | 1)  ß [95% *CI*] | 2)  ß [95% *CI*] | 3)  ß [95% *CI*] | 4)  ß [95% *CI*] | 5)  ß [95% *CI*] | 6)  ß [95% *CI*] |
| CES-D | **.483****  **[.339, .539]** | **.510****  **[.360, .565]** | **.506****  **[.359, .561]** | **.559****  **[.404, .612]** | **.532****  **[.379, .587]** | **.545****  **[.390, .599]** |
| CAPE-POS | -.043  [-.206, .089] | -.052  [-.222, .082] | -.020  [-.178, .123] | -.040  [-.209, .099] | -.042  [-.212, .097] | -.038  [-.209, .105] |
| internality | **-.268****  **[-.236, -.104]** | - | - | -.101  [-.128, .004] | - | **-** |
| stability | - | **-.171***  **[-.208, -.048]** | - | - | .055  [-.029, .090] | **-** |
| globality | - | - | **-.211****  **[-.168, -.057]** | - | - | -.018  [-.063, .045] |
| ΔR² for attribution predictor | .068 | .028 | .043 | .010 | .003 | .000 |
| adjusted R^2^ | .340 | .299 | .314 | .281 | .274 | .271 |
| F | **43.860**** | **36.526**** | **39.216**** | **33.502**** | **32.377**** | **31.952**** |

*Note.* *n*=251. Multiple linear regression analyses for each attributional dimension as independent variable with amotivation as dependent variable.

ß = standardized coefficient estimate. *CI* = 95% confidence interval in square brackets. CES-D = Center for Epidemiologic Studies-Depression Scale. CAPE-POS = Community Assessment of Psychic Experiences, positive symptoms subscale. MAP-SR = Motivation and Pleasure Scale-Self-Report.

For the interpretation of the regression analyses, the Bonferroni corrected level of significance .05/6= .0083 has been implemented.

**** p* < .0083.; ** *p* ≤ .001**.

**References**

1. Llerena K, Park SG, McCarthy JM, Couture SM, Bennett ME, Blanchard JJ. The Motivation and Pleasure Scale-Self-Report (MAP-SR): Reliability and validity of a self-report measure of negative symptoms. Compr Psychiatry [Internet]. 2013;54(5):568–74. Available from: http://dx.doi.org/10.1016/j.comppsych.2012.12.001

2. Peterson C, Semmel A, von Baeyer C, Abramson LY, Metalsky GI, Seligman MEP. The Attributional Style Questionnaire. Cognit Ther Res [Internet]. 1982 Sep;6(3):287–99. Available from: http://link.springer.com/10.1007/BF01173577

3. Poppe P, Stiensmeier-Pelster J, Pelster A. Attributionsstilfragebogen für Erwachsene: ASF-E. Hogrefe; 2005.

4. Stefanis NC, Hanssen M, Smirnis NK, Avramopoulos DA, Evdokimidis IK, Stefanis CN, et al. Evidence that three dimensions of psychosis have a distribution in the general population. Psychol Med. 2002;32(2):347–58.

5. Radloff LS. The CES-D Scale: A Self-Report Depression Scale for Research in the General Population. Appl Psychol Meas. 1977;1(3):385–401.

6. Cohen J. Statistical Power Analysis for the Behavioral Sciences. 2nd ed. Hillsdale, NJ: Lawrence Erlbaum Associates Publishers; 1988.
